# Association of pulse wave velocity with total lung capacity: A cross-sectional analysis of the BOLD London study

**DOI:** 10.1016/j.rmed.2015.10.016

**Published:** 2015-12

**Authors:** André F.S. Amaral, Jaymini Patel, Louisa Gnatiuc, Meinir Jones, Peter G.J. Burney

**Affiliations:** Respiratory Epidemiology, Occupational Medicine and Public Health, National Heart and Lung Institute, Imperial College, London, UK

**Keywords:** Lung function, Total lung capacity, Pulmonary restriction, Airflow obstruction, Cardiovascular disease, Pulse wave velocity

## Abstract

**Background:**

Low lung function, measured using spirometry, has been associated with mortality from cardiovascular disease, but whether this is explained by airflow obstruction or restriction is a question that remains unanswered.

**Objectives:**

To assess the association of total lung capacity (TLC), forced vital capacity (FVC) and forced expiratory volume in 1 s (FEV1) with several cardio-metabolic and inflammatory markers.

**Methods:**

In the follow up of the Burden of Lung Disease (BOLD) study in London, acceptable post-bronchodilator spirometric, pulse rate, pulse wave velocity and blood pressure data were obtained from 108 participants. Blood samples for measurement of cardio-metabolic and inflammatory markers were also collected from these participants. Association of lung function and volume with the different biomarkers was examined in multivariable linear regression models adjusted for potential confounders.

**Results:**

Following adjustment for age, sex, height, and ethnicity, TLC (adjusted coefficient = −1.53; 95% CI: −2.57, −0.49) and FVC (adjusted coefficient = −2.66; 95% CI: −4.98, −0.34) were inversely associated with pulse wave velocity, and further adjustment for smoking status, pack-years and body mass index (BMI) did not materially change these results. FEV1 was inversely associated with systolic blood pressure, and adjustment for smoking status, pack-years and BMI made this association stronger (adjusted coefficient = −9.47; 95% CI: −15.62, −3.32).

**Conclusion:**

The inverse association of pulse wave velocity, which is a marker of cardiovascular disease, with TLC suggests that the association of the former with low FVC is independent of airflow obstruction. The association between FEV1 with systolic blood pressure after adjustment for FVC suggests an association with airflow obstruction rather than with restricted spirometry.

## Highlights

• TLC is inversely associated with pulse wave velocity (i.e. arterial stiffness).
• FVC, which is a proxy for TLC, is also inversely associated with pulse wave velocity.
• Systolic blood pressure is inversely associated with FEV1.

Low lung function has been associated with increased mortality, including mortality from cardiovascular disease [1], [2], [3], [4]. Although many authors have linked this to airflow obstruction, because of the connection with forced expiratory volume in 1 s (FEV1), those that have looked separately at forced vital capacity (FVC) and at these two measures analysed together have shown that the strong association is between mortality and FVC, not airflow obstruction [5]. Similarly, several of the co-morbidities associated with cardiovascular diseases (e.g. impaired glucose tolerance, hypertension, and obesity) have been shown to associate with low FVC [6], [7]. However, interpretation is complicated by the increased residual volume in more severe obstruction which may also lead to a reduction in FVC.

Few population-based studies have specifically tested the association of cardio-metabolic and inflammatory markers with lung function. The Whitehall II Study and the Caerphilly Prospective Study reported an inverse association between pulse wave velocity and FVC and/or FEV1 [8], [9]. The US Third National Health and Nutrition Examination Survey (NHANES III) reported an inverse association between pulse pressure and FEV1 [10]. The Multiethnic Study of Atherosclerosis (MESA) reported that both FEV1 and FVC are positively associated with small artery elasticity, and inversely associated with serum markers of endothelial dysfunction and inflammation such as intercellular adhesion molecule 1 (ICAM-1), fibrinogen, C-reactive protein (CRP), and interleukin 6 (IL-6) [11]. The NHANES III and the Korean National Health and Nutrition Examination Survey (KNHANES) have both reported an inverse association between glycated haemoglobin (HbA1c) and both FEV1 and FVC [12], [13].

None of the studies mentioned above has looked at the association of cardio-metabolic or inflammatory markers with total lung capacity (TLC), which would be relevant to the interpretation of findings carried out using FVC, especially when there is some evidence suggesting that the TLC might be a better predictor of mortality and hospitalisation than the FVC in older people [14].

The main aim of the present analysis was to assess the association of TLC with several cardio-metabolic and inflammatory markers, as surrogate outcomes for cardiovascular disease. It was also our aim to examine whether FVC is a good substitute of TLC in predicting the levels of these markers. To achieve our aims, we have measured the associations of the different cardio-metabolic and inflammatory markers with TLC, FVC and FEV1 in participants who took part in the follow up of the UK arm of the Burden of Obstructive Lung Disease (BOLD) study, and tested whether these simply reflect a common association with smoking and body mass index (BMI).

## 1 Methods

### 1.1 Study participants

**Fig. 1:**
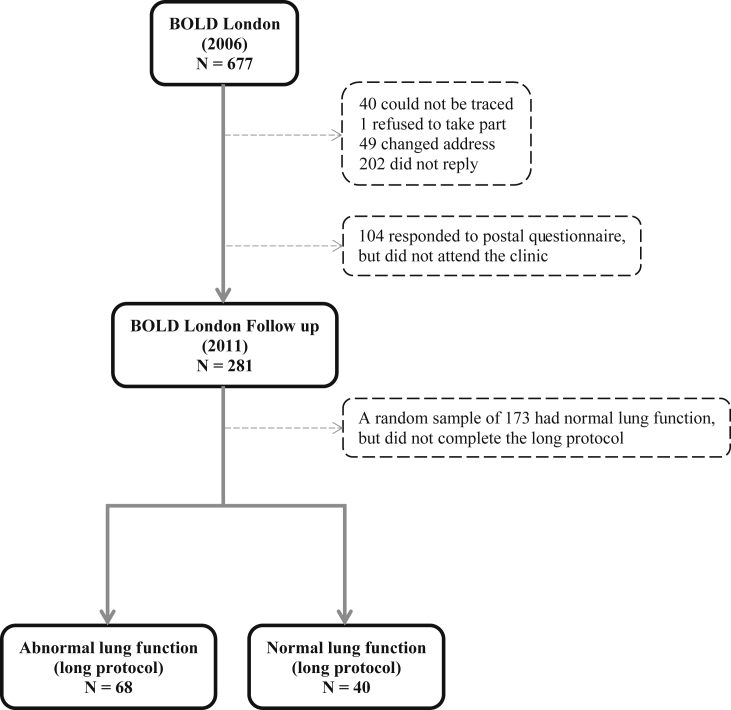
Participants from the Burden of Lung Disease London follow-up included in this report.

The design and rationale for the multicentre BOLD study and its UK arm have been reported elsewhere [15], [16]. Briefly, in 2006 non-institutionalised adults aged 40 years and older were sampled from the lists of three general practices in the Borough of Hammersmith and Fulham, London, and invited to take part in the study. This selection process led to 677 participants in the study. In 2011, 636 (94%) participants, who agreed in 2006 to take part in a follow up, were again approached and 281 (42%) responded to a postal questionnaire and attended the clinic for further lung function measurements (Fig. 1). These were split into two groups based on their characteristics in 2006: 1) participants at high risk of an abnormal lung function [i.e. those who have had an FEV1/FVC < lower limit of normal (LLN) (n = 34), FVC < LLN (n = 23), or who have reported an history of either emphysema (n = 6) or past tuberculosis (n = 5)]; 2) all other eligible participants. The first group (n = 68) and a random sample of the second group (n = 40), stratified by age, sex and ethnicity, completed the same long protocol. The rest of participants completed a short protocol of just the modified questionnaire and pre- and post-bronchodilator spirometry (n = 173). This report is based on data just from participants who completed the long version of the BOLD protocol. This comprehensive protocol involved the collection of information on basic demographics, smoking habits, history of respiratory symptoms and co-morbidities, use of respiratory medication, risk factors for chronic obstructive pulmonary disease, healthcare utilisation, occupational exposures [15], and diet, the collection of blood for measurement of biomarkers, spirometry for measurement of lung function, and body plethysmography for measurement of lung volumes. Ethical approval for the follow up study was obtained from the National Research Ethics Service (Oxfordshire REC C), and all participants provided written informed consent.

### 1.2 Cardio-metabolic, inflammatory and other serum markers

Pulse rate, pulse wave velocity, and systolic and diastolic blood pressures were measured using a Vicorder (Skidmore Medical, Bristol, UK). Blood was collected for routine measurement of white blood cells and HbA1c, and for measurement of other serum markers. Cytokines and inflammatory markers [CRP, interleukin 8 (IL-8), IL-6, tumour necrosis factor alpha (TNF-alpha), myeloperoxidase (MPO)], markers of vascular injury [vascular cell adhesion protein 1 (VCAM-1), ICAM-1, serum amyloid A (SAA)], proteinases and anti-proteases [matrix metalloproteinase 3 (MMP-3), matrix metalloproteinase 9 (MMP-9) and tissue inhibitor of metalloproteinase 1 (TIMP-1)], were measured in serum samples using a MULTI-ARRAY^®^ assay system according to the manufacturer's instructions (Meso Scale Discovery, Gaithersburg, MD, USA).

### 1.3 Lung measurements

TLC was measured at Charing Cross Hospital using body plethysmography following the ATS/ERS criteria [17]. FEV1 and FVC were measured in the same facility by a trained member of the BOLD team using the ndd EasyOne Spirometer (ndd Medizintechnik AG, Zurich, Switzerland), before and 15 min after administration of salbutamol (200 μg) from a metred dose inhaler through a spacer. The BOLD Pulmonary Function Reading Centre reviewed each spirogram and assigned them a quality score based on acceptability and reproducibility criteria from the American Thoracic Society (ATS) and European Respiratory Society (ERS) [18].

### 1.4 Statistical methods

Statistical analyses were performed using Stata V.12.1 (StataCorp LP, College Station, TX). The set of commands for surveys (‘svy’) were used to take into account the sampling design and correct for the effect of clustering. To examine the association of each of the cardio-metabolic and inflammatory markers with each of the lung function measurements, multivariable linear regression models, allowing for sample weights derived by estimating the probability of selection, were used. These models were adjusted for age, sex, height, and ethnicity. The models for FEV1 (or FVC) were also adjusted for FVC (or FEV1). All models were further adjusted for smoking status and pack-years and BMI. Results were considered statistically significant when *P* was equal or less than 0.05.

## 2 Results

Of the 108 participants who responded to the postal questionnaire and attended the clinic for further lung function measurements, 55.7% were females, 84% were white, and their median age was 59 years. The median TLC, FVC, and FEV1 of these participants was 5.65 L, 3.32 L, and 2.46 L, respectively. A description of the characteristics of the participants in this study is presented in Table 1.

Table 2, Table 3, Table 4 show the unadjusted and adjusted regression coefficients, with 95% confidence intervals (CIs), for the association of TLC, FVC and FEV1 with the different cardio-metabolic and inflammatory markers.

**Fig. 2:**
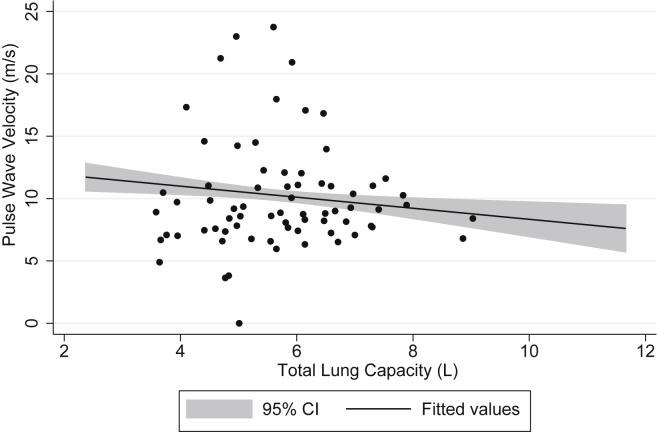
Association of pulse wave velocity with total lung capacity, adjusted for age, sex, ethnicity, smoking pack-years, and body mass index.

**Fig. 3:**
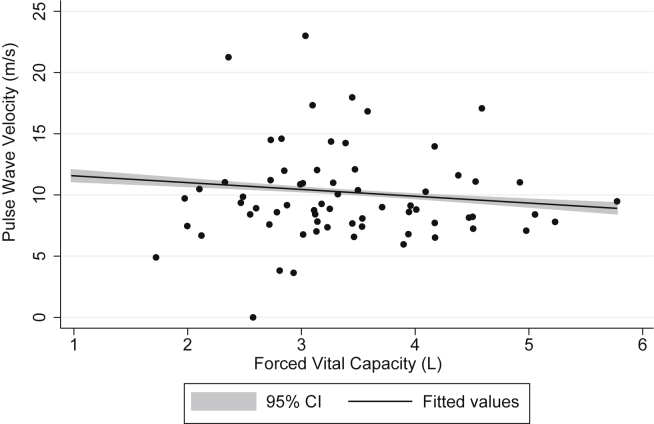
Association of pulse wave velocity with forced vital capacity, adjusted for age, sex, ethnicity, smoking pack-years, and body mass index.

After adjustment for age, sex, height, and ethnicity (and FEV1 in models for FVC), both TLC (adjusted coefficient = −1.53; 95% CI: −2.57, −0.49) and FVC (adjusted coefficient = −2.66; 95% CI: −4.98, −0.34) were inversely associated with pulse wave velocity. Further adjustment for smoking status, pack-years and BMI did not materially change these results (Table 2, Table 3; Fig. 2, Fig. 3). Although FVC was highly correlated with TLC (correlation coefficient = 0.77, *P* < 0.001), the model for the association of pulse wave velocity with TLC (r-squared = 0.22) had a better fit than the one for FVC (r-squared = 0.10). TLC was associated with HbA1c (unadjusted coefficient = −0.09; 95% CI: −0.17, −0.001), but after adjustment this association was no longer significant (Table 2). FVC was associated with systolic blood pressure (unadjusted coefficient = −5.20; 95% CI: −9.30, −1.10) and MMP-3 (unadjusted coefficient = 2.64; 95% CI: 0.89, 4.39), but after adjustment for confounders these associations became non-significant (Table 3). There was a borderline significant association of FVC with HbA1c after adjustment for age, sex, height, ethnicity, FEV1 and smoking (adjusted coefficient = −0.28; 95% CI: −0.58, 0.01), but this disappeared after including BMI in the model (adjusted coefficient = −0.09; 95% CI: −0.40, 0.21). FVC and TLC were not significantly associated with any other cardio-metabolic or inflammatory marker measured.

FEV1 was inversely associated with systolic blood pressure (unadjusted coefficient = −7.67; 95% CI: −12.14, −3.20), even after adjustment for age, sex, height, ethnicity and FVC (adjusted coefficient = −7.02; 95% CI: −12.58, −1.46), smoking status and pack-years (adjusted coefficient = −8.71; 95% CI: −14.77, −2.64) and BMI (adjusted coefficient = −9.47; 95% CI: −15.62, −3.32). FEV1 was not significantly associated with any other cardio-metabolic marker (Table 4).

In sensitivity analyses with fully adjusted models but excluding participants with abnormal lung function, the associations of pulse wave velocity with TLC (adjusted coefficient = −1.70; 95% CI: −3.78, 0.39) and FVC (adjusted coefficient = −5.17; 95% CI: −16.98, 6.65) were strengthened but not significant, and the association of systolic blood pressure with FEV1 (adjusted coefficient = −26.97; 95% CI: −43.97, −9.98) was strengthened and significant.

## 3 Discussion

In this population-based study, we observed little association between the several cardio-metabolic markers and measures of lung function and volume. However, after adjustment for confounders, FEV1 was inversely associated with systolic blood pressure, and both TLC and FVC were inversely associated with pulse wave velocity, a marker of arterial stiffness. This condition is an important risk factor for cardiovascular disease and mortality even among apparently healthy people, and has been associated with atherosclerosis and left ventricular hypertrophy, which is a risk factor for congestive heart failure [19], [20], [21].

The strengths of this study are the use of a standardised questionnaire for assessment of risk factors and high quality protocol for spirometry, the use of post- instead of pre-bronchodilator spirometric measurements, and the use of body plethysmography to measure TLC, which is rare in epidemiological studies. In people with severe obstruction a significant quantity of air may remain trapped during the spirometric forced expiratory manoeuvre, increasing the residual volume and decreasing the FVC, which would incorrectly suggest the presence of restriction. The measurement of TLC using plethysmography, which is independent of the forced expiratory manoeuvre, allows identification of true cases of lung restriction. Body plethysmography is a highly informative method to assess airway obstruction and lung volumes [22], but because it needs to be carried out in a clinical setting and it is technically demanding it is not commonly used in large studies. The main limitations are: 1) the small number of subjects in the analysis, as compared to the initial sample in BOLD London; and 2) the non-longitudinal nature of the present analysis, which prevents us from drawing strong conclusions in terms of temporality. This small sample size was partly by design and partly the result of a very itinerant population and a general lack of interest for surveys in large cities, such as London, which both reduce response rates. We used sampling weights to mitigate the effects of cohort attrition and to allow for the oversampling of those with abnormal spirometry at the first visit.

To our knowledge this is the first study to report an inverse association between TLC and pulse wave velocity. However, reduced TLC has been previously associated with overall mortality in an Italian study of elderly participants without airway obstruction [14].

The inverse association of FVC with pulse wave velocity agrees with previous reports, such as those from the Whitehall II Study [9], the Caerphilly Prospective Study [8], and MESA (used artery elasticity instead of pulse wave velocity) [11]. Our finding of an inverse association of FEV1 with systolic blood pressure is consistent with previous reports, such as those from the Cardiovascular Health Study [23], NHANES III (used pulse rate instead of systolic blood pressure) [10], Coronary Artery Risk Development in Young Adults [7], and Prospective Urban and Rural Epidemiological Study in South Africa [24].

After adjustment for several confounders, we found no significant associations between TLC or FVC or FEV1 with markers of inflammation, vascular injury or HbA1c. This is in contrast with results from MESA, which reported inverse associations of both FVC and FEV1 with ICAM-1, CRP, and IL-6 [11], and from NHANES III [12] and KNHANES which have reported an inverse association between the two spirometric measures and HbA1c [13]. However, our study is smaller than the previous ones and random error in such measurements may explain the lack of significant associations with the markers of inflammation, vascular injury and insulin resistance.

The lack of evidence for a strong association of lung function with these markers suggests that the association of lung function with systolic blood pressure and arterial stiffness is not mediated by either inflammation or insulin resistance. However, this does not exclude the possibility that other cardio-metabolic or inflammatory makers may be involved in the underlying mechanisms that link lung function and arterial stiffness or that a low lung function may lead to a high systolic blood pressure and pulse wave velocity. In a population-based study with adults free of coronary heart disease and stroke at baseline, low peak expiratory flow was reported as a good predictor of carotid atherosclerosis at four years of follow up [25]. The Caerphilly Prospective Study has reported that early lung function measurements compared to later life measurements are strong predictors of arterial stiffness [8].

In conclusion, our findings show that reduced TLC and FVC, which correlate to each other very well, are associated with higher pulse wave velocity, a marker of atherosclerotic changes, but the fit of the model using TLC is much better. This supports the impression that the association between mortality and a low FVC is reflecting a more basic association with a low TLC and may explain why as populations age or become sicker the TLC is better predictor of future mortality [14]. This also adds to the evidence that the relationship between low lung function and cardiovascular disease is largely independent of airway obstruction, although we found FEV1 to inversely associate with systolic blood pressure. Large longitudinal studies are needed to establish whether or not low lung function precedes cardiovascular disease and mortality.

## Author contribution

Conception and design: PGJB; Analysis and interpretation: AFSA, JP, MJ, PGJB; Drafting the manuscript: AFSA; Critical revision of the manuscript: AFSA, JP, LG, MJ, PGJB.

## Figures and Tables

**Table 1:** Characteristics of the participants in the BOLD London Follow up who completed the long protocol.

	Abnormal lung function (N = 68)	Normal lung function (N = 40)	Total (N = 108)
Age (years), median (IQR)	58 (51–67)	60.5 (51–65)	59 (51–67)
Sex (%)			
Females	40 (59)	23 (58)	63 (58)
Males	28 (41)	17 (42)	45 (42)
Ethnicity (%)			
White	49 (72)	36 (90)	85 (79)
Other	19 (28)	4 (10)	23 (21)
Height (cm), median (IQR)	165 (159–171)	168 (159–174)	168 (161–175)
Body mass index (kg/m^2^), median (IQR)	25.7 (23.1–31.6)	27.8 (24.4–30.7)	26.4 (23.6–29.8)
Pack-years of smoking, median (IQR)	12.6 (0–31.4)	1.18 (0–13.5)	4.7 (0–20.6)
TLC (L), median (IQR)	5.62 (4.84–6.46)	5.72 (4.77–6.59)	5.65 (4.76–6.43)
FVC (L), median (IQR)	3.22 (2.54–3.70)	3.22 (2.91–4.07)	3.22 (2.72–4.53)
FVC % predicted, median (IQR)	83.5 (75.1–102.2)	98.1 (89.9–105.0)	92.0 (78.1–104.0)
FEV1 (L/s), median (IQR)	2.05 (1.60 2.50)	2.50 (2.24–3.08)	2.46 (2.04–3.03)
FEV1% predicted, median (IQR)	73.4 (63.8–82.7)	91.2 (81.8–97.7)	79.4 (69.9–91.7)
Pulse rate (beats/min), median (IQR)	70 (64–76)	68 (61–74)	68 (63–76)
Pulse wave velocity (m/s), median (IQR)	9 (7–11)	9 (8–12)	9 (8–11)
Systolic blood pressure (mm Hg), median (IQR)	140 (127–151)	139 (130–146)	140 (129–148)
Diastolic blood pressure (mm Hg), median (IQR)	78 (72–81)	76 (70–82)	78 (72–82)
White blood cell count (x10^9^/L), median (IQR)	6.35 (5.4–7.3)	5.6 (5–6.9)	6.0 (5.1–7.3)
HbA1c (%), median (IQR)	5.65 (5.4–5.9)	5.6 (5.3–5.7)	5.6 (5.4–5.8)
C-reactive protein (μg/mL), median (IQR)	1.19 (0.41–3.17)	0.82 (0.36–2.61)	1.01 (0.36–2.63)
IL-8 (pg/mL), median (IQR)	4.99 (3.80–6.74)	5.24 (4.05–6.99)	5.15 (3.89–6.99)
IL-6 (pg/mL), median (IQR)	1.03 (0.70–1.43)	0.98 (0.63–1.81)	1.02 (0.71–1.69)
TNF-alpha (pg/mL), median (IQR)	3.44 (2.37–4.44)	3.69 (2.52–5.46)	3.62 (2.43–5.29)
MPO (ng/mL), median (IQR)	0.35 (0.26–0.54)	0.32 (0.20–0.63)	0.35 (0.24–0.55)
VCAM-1 (ng/mL), median (IQR)	226.0 (186.4–272.6)	206.0 (181.0–224.4)	214.5 (181.0–262.6)
ICAM-1 (ng/mL), median (IQR)	146.6 (120.0–193.3)	134.7 (112.0–156.6)	139.0 (112.8–167.2)
SAA (μg/mL), median (IQR)	0.68 (0.41–1.20)	0.67 (0.45–1.03)	0.68 (0.41–1.11)
MMP-3 (ng/mL), median (IQR)	11.42 (7.44–16.77)	12.10 (7.34–15.17)	11.99 (7.69–18.19)
MMP-9 (ng/mL), median (IQR)	48.46 (32.92–69.33)	40.65 (26.02–57.88)	45.43 (29.88–66.05)
TIMP-1 (ng/mL), median (IQR)	28.38 (22.06–34.83)	26.43 (23.17–31.68)	26.90 (21.73–33.51)

IQR, interquartile range. TLC, total lung capacity. FVC, forced vital capacity. FEV1, forced expiratory volume in 1 s. HbA1c, glycated haemoglobin. IL-8, interleukin 8. IL-6, interleukin 6. TNF-alpha, tumour necrosis factor alpha. MPO, myeloperoxidase. VCAM-1, vascular cell adhesion protein 1. ICAM-1, intercellular adhesion molecule 1. SAA, serum amyloid A. MMP-3, matrix metalloproteinase 3. MMP-9, matrix metalloproteinase 9. TIMP-1, tissue inhibitor of metalloproteinase 1.

**Table 2:** Association of cardio-metabolic markers with total lung capacity (TLC).

Cardio-metabolic markers	Unadjusted coefficient (95% CI)	P	Adjusted coefficient* (95% CI)	P	Adjusted coefficient** (95% CI)	P	Adjusted coefficient*** (95% CI)	P
Pulse rate (beats/min)	−0.54 (−2.47, 1.40)	0.58	−1.02 (−4.16, 2.13)	0.52	−0.79 (−3.96, 2.39)	0.62	−0.45 (−3.32, 2.42)	0.75
Pulse wave velocity (m/s)	−0.27 (−1.01, 0.47)	0.47	−1.53 (−2.57, −0.49)	<0.001	−1.49 (−2.45, −0.52)	<0.001	−1.29 (−2.19, −0.39)	0.01
Systolic blood pressure (mm Hg)	−2.02 (−4.66, 0.62)	0.13	−1.81 (−4.90, 1.28)	0.25	−1.84 (−5.00, 1.31)	0.25	−1.00 (−4.63, 2.63)	0.59
Diastolic blood pressure (mm Hg)	0.88 (−0.80, 2.55)	0.30	−0.10 (−2.14, 1.94)	0.92	−0.20 (−2.24, 1.84)	0.85	0.26 (−1.91, 2.42)	0.81
White blood cell count (x10^9^/L)	−0.05 (−0.37, 0.28)	0.78	−0.15 (−0.59, 0.30)	0.52	−0.15 (−0.59, 0.30)	0.51	0.04 (−0.41, 0.50)	0.85
HbA1c (%)	−0.09 (−0.17, −0.001)	0.05	−0.08 (−0.23, 0.06)	0.27	−0.08 (−0.22, 0.06)	0.24	−0.03 (−0.16, 0.10)	0.65
C-reactive protein (μg/mL)	−0.13 (−0.62, 0.36)	0.61	−0.06 (−0.74, 0.62)	0.86	−0.09 (−0.79, 0.61)	0.80	0.31 (−0.31, 0.93)	0.32
IL-8 (pg/mL)	0.23 (−0.29, 0.75)	0.39	0.38 (−0.32, 1.09)	0.29	0.37 (−0.35, 1.09)	0.31	0.28 (−0.34, 0.90)	0.37
IL-6 (pg/mL)	−0.25 (−0.86, 0.35)	0.41	−0.24 (−0.70, 0.21)	0.29	−0.26 (−0.74, 0.21)	0.28	−0.10 (−0.52, 0.31)	0.62
TNF-alpha (pg/mL)	0.28 (−0.14, 0.70)	0.18	0.30 (−0.37, 0.98)	0.38	0.31 (−0.37, 0.99)	0.37	0.15 (−0.44, 0.74)	0.61
MPO (ng/mL)	0.02 (−0.02, 0.06)	0.33	−0.03 (−0.11, 0.05)	0.50	−0.03 (−0.11, 0.05)	0.48	0.00 (−0.07, 0.07)	1.00
VCAM-1 (ng/mL)	−0.58 (−16.16, 14.99)	0.94	−1.73 (−23.28, 19.82)	0.87	−1.89 (−23.38, 19.61)	0.86	4.35 (−15.05, 23.75)	0.66
ICAM-1 (ng/mL)	5.31 (−2.60, 13.23)	0.19	9.80 (−2.46, 22.06)	0.12	9.23 (−2.94, 21.39)	0.14	8.36 (−1.90, 18.61)	0.11
SAA (μg/mL)	0.16 (−0.35, 0.66)	0.54	0.24 (−0.32, 0.81)	0.40	0.24 (−0.33, 0.82)	0.40	0.32 (−0.18, 0.82)	0.20
MMP-3 (ng/mL)	1.06 (−0.71, 2.84)	0.24	−0.78 (−3.55, 1.99)	0.58	−0.79 (−3.57, 1.98)	0.57	−0.35 (−2.61, 2.54)	0.98
MMP-9 (ng/mL)	4.08 (−3.67, 11.82)	0.30	1.67 (−10.51, 13.85)	0.79	1.59 (−10.69, 13.87)	0.80	1.92 (−9.91, 13.75)	0.75
TIMP-1 (ng/mL)	1.03 (−0.56, 2.62)	0.20	1.18 (−0.90, 3.25)	0.26	1.18 (−0.88, 3.23)	0.26	1.43 (−0.44, 3.23)	0.13

*Adjusted for age, sex, height, and ethnicity. **Adjusted for age, sex, height, ethnicity, smoking status and pack-years. ***Adjusted for age, sex, ethnicity, smoking status, pack-years, and body mass index.

**Table 3:** Association of cardio-metabolic markers with forced vital capacity (FVC).

Cardio-metabolic markers	Unadjusted coefficient (95% CI)	P	Adjusted coefficient* (95% CI)	P	Adjusted coefficient** (95% CI)	P	Adjusted coefficient*** (95% CI)	P
Pulse rate (beats/min)	0.16 (−2.50, 2.82)	0.91	−1.51 (−8.20, 5.18)	0.65	−1.92 (−8.74, 4.90)	0.58	−0.82 (−7.81, 6.17)	0.82
Pulse wave velocity (m/s)	−0.28 (−1.47, 0.91)	0.64	−2.66 (−4.98, −0.34)	0.03	−2.98 (−5.31, −0.65)	0.01	−2.85 (−5.59, −0.11)	0.04
Systolic blood pressure (mm Hg)	−5.20 (−9.30, −1.10)	0.01	−0.30 (−8.06, 7.45)	0.94	−0.10 (−7.58, 7.37)	0.98	5.43 (−1.90, 12.76)	0.14
Diastolic blood pressure (mm Hg)	0.07 (−1.99, 2.12)	0.95	−2.18 (−7.40, 3.03)	0.41	−1.30 (−6.53, 3.93)	0.62	2.59 (−2.67, 7.84)	0.33
White blood cell count (x10^9^/L)	−0.03 (−0.46, 0.39)	0.88	0.48 (−0.46, 1.42)	0.31	0.36 (−0.65, 1.37)	0.48	0.66 (−0.36, 1.67)	0.20
HbA1c (%)	−0.12 (−0.28, 0.04)	0.15	−0.23 (−0.53, 0.06)	0.12	−0.28 (−0.58, 0.01)	0.06	−0.09 (−0.40, 0.21)	0.54
C-reactive protein (μg/mL)	−0.36 (−1.03, 0.31)	0.29	−0.95 (−2.74, 0.85)	0.30	−1.23 (−3.00, 0.54)	0.17	0.52 (−0.71, 1.75)	0.40
IL-8 (pg/mL)	−0.20 (−0.99, 0.60)	0.63	−0.99 (−2.55, 0.57)	0.21	−1.12 (−2.75, 0.35)	0.18	−1.05 (−2.67, 0.57)	0.20
IL-6 (pg/mL)	−0.48 (−1.34, 0.39)	0.28	−0.95 (−2.12, 0.21)	0.11	−1.04 (−2.43, 0.35)	0.14	−0.61 (−1.62, 0.40)	0.24
TNF-alpha (pg/mL)	−0.18 (−0.81, 0.44)	0.56	−0.71 (−2.28, 0.86)	0.37	−0.77 (−2.36, 0.82)	0.34	−1.10 (−2.43, 0.23)	0.11
MPO (ng/mL)	0.00 (−0.06, 0.07)	0.90	−0.13 (−0.30, 0.03)	0.10	−0.13 (−0.28, 0.02)	0.09	−0.09 (−0.23, 0.05)	0.19
VCAM-1 (ng/mL)	−13.76 (−36.29, 8.76)	0.23	−14.23 (−67.21, 38.76)	0.60	−15.62 (−67.63, 36.38)	0.55	10.93 (−24.47, 46.34)	0.54
ICAM-1 (ng/mL)	−1.66 (−14.51, 11.20)	0.80	13.94 (−14.87, 42.74)	0.34	8.67 (−20.88, 38.21)	0.56	12.34 (−13.71, 38.39)	0.35
SAA (μg/mL)	0.13 (−0.51, 0.76)	0.69	0.25 (−0.54, 1.05)	0.53	0.44 (−0.73, 0.60)	0.46	0.76 (−0.20, 1.72)	0.12
MMP-3 (ng/mL)	2.64 (0.89, 4.39)	<0.001	3.94 (−1.29, 9.17)	0.14	4.23 (−1.14, 9.60)	0.12	4.16 (−1.26, 9.57)	0.13
MMP-9 (ng/mL)	2.76 (−6.21, 11.73)	0.54	17.61 (−11.44, 46.65)	0.23	15.86 (−15.67, 47.39)	0.32	17.10 (−16.59, 50.79)	0.32
TIMP-1 (ng/mL)	0.14 (−2.36, 2.63)	0.91	3.80 (−3.39, 11.00)	0.30	2.68 (−4.18, 9.54)	0.44	3.61 (−2.62, 9.83)	0.25

*Adjusted for age, sex, height, ethnicity, and forced expiratory volume in 1 s (FEV1). **Adjusted for age, sex, height, ethnicity, FEV1, smoking status and pack-years. ***Adjusted for age, sex, ethnicity, FEV1, smoking status, pack-years, and body mass index.

**Table 4:** Association of cardio-metabolic markers with forced expiratory volume in 1 s (FEV1).

Cardio-metabolic markers	Unadjusted coefficient (95% CI)	P	Adjusted coefficient^∗^ (95% CI)	P	Adjusted coefficient** (95% CI)	P	Adjusted coefficient*** (95% CI)	P
Pulse rate (beats/min)	0.25 (−2.84, 3.34)	0.87	−0.08 (−6.62, 6.46)	0.98	0.81 (−6.57, 8.18)	0.83	0.33 (−7.30, 7.97)	0.93
Pulse wave velocity (m/s)	−0.11 (−1.38, 1.16)	0.86	1.80 (−0.54, 4.14)	0.13	2.07 (−0.36, 4.51)	0.09	2.12 (−0.37, 4.60)	0.09
Systolic blood pressure (mm Hg)	−7.67 (−12.14, - 3.20)	<0.001	−7.02 (−12.58, −1.46)	0.01	−8.71 (−14.77, −2.64)	0.01	−9.47 (−15.62, −3.32)	<0.001
Diastolic blood pressure (mm Hg)	0.01 (−2.29, 2.31)	0.99	−1.38 (−6.41, 3.65)	0.59	−3.54 (−8.85, 1.77)	0.19	−4.17 (−9.80, 1.46)	0.14
White blood cell count (x10^9^/L)	−0.30 (−0.81, 0.21)	0.25	−0.66 (−1.64, 0.32)	0.19	−0.43 (−1.61, 0.76)	0.48	−0.72 (−1.92, 0.49)	0.24
HbA1c (%)	−0.12 (−0.28, 0.04)	0.13	0.001 (−0.24, 0.24)	0.99	0.10 (−0.14, 0.34)	0.40	−0.04 (−0.28, 0.19)	0.71
C-reactive protein (μg/mL)	−0.63 (−1.46, 0.20)	0.14	0.40 (−1.69, 2.50)	0.70	0.13 (−0.08, 3.33)	0.23	0.32 (−1.55, 2.18)	0.74
IL-8 (pg/mL)	−0.15 (−1.01, 0.70)	0.72	−0.69 (−2.45, 1.07)	0.44	−0.56 (−2.56, 1.45)	0.58	−0.39 (−2.57, 1.79)	0.72
IL-6 (pg/mL)	−0.43 (−1.14, 0.28)	0.23	0.14 (−0.55, 0.83)	0.69	0.46 (−0.36, 1.27)	0.27	0.38 (−0.41, 1.17)	0.34
TNF-alpha (pg/mL)	−0.30 (−1.05, 0.45)	0.43	−0.51 (−2.06, 1.04)	0.51	−0.65 (−2.49, 1.20)	0.49	−0.18 (−2.17, 1.82)	0.86
MPO (ng/mL)	0.03 (−0.04, 0.09)	0.41	0.07 (−0.08, 0.22)	0.33	0.07 (−0.08, 0.23)	0.36	0.07 (−0.09, 0.23)	0.38
VCAM-1 (ng/mL)	−23.72 (−49.17, 1.73)	0.07	−18.85 (−85.11, 47.41)	0.57	−14.61 (−81.68, 52.47)	0.67	−28.21 (−95.12, 38.69)	0.40
ICAM-1 (ng/mL)	−8.23 (−23.95, 7.49)	0.30	−27.73 (−60.50, 5.04)	0.10	−13.96 (−48.14, 20.23)	0.42	−19.93 (−56.76, 16.90)	0.29
SAA (μg/mL)	0.01 (−0.67, 0.69)	0.97	0.57 (−0.43, 1.58)	0.26	0.39 (−0.32, 1.11)	0.28	0.10 (−0.57, 0.76)	0.78
MMP-3 (ng/mL)	1.49 (−0.37, 3.35)	0.12	−4.73 (−10.58, 1.12)	0.11	−5.51 (−11.63, 0.60)	0.08	−4.82 (−11.03, 1.39)	0.13
MMP-9 (ng/mL)	−4.96 (−12.79, 2.87)	0.21	−24.85 (−58.59, 8.88)	0.15	−21.71 (−59.19, 15.77)	0.25	−21.48 (−60.15, 17.19)	0.27
TIMP-1 (ng/mL)	−1.29 (−3.59, 1.01)	0.27	−2.81 (−7.79, 2.17)	0.27	−1.11 (−5.74, 3.52)	0.63	−2.12 (−7.03, 2.79)	0.39

^∗^Adjusted for age, sex, height, ethnicity, and forced vital capacity (FVC).**Adjusted for age, sex, height, ethnicity, FVC, smoking status and pack-years. ***Adjusted for age, sex, ethnicity, FVC, smoking status, pack-years, and body mass index.
